# Understanding the Prospective Acceptability of a Fall Prevention Exercise App Among Canadians Aged 50 Years and Older Living in the Community: Mixed Methods Study

**DOI:** 10.2196/95812

**Published:** 2026-08-13

**Authors:** Maria Fernanda Fuentes Diaz, Kathryn May Sibley, Chinasa Obiageri Trinitta Ugwuegbulem, Kim Delbaere, Danielle R Bouchard

**Affiliations:** 1 Cardiometabolic Exercise and Lifestyle Laboratory, Faculty of Kinesiology, University of New Brunswick, 90 Mckay Drive, Fredericton, NB, E3B 5A3, Canada, 1 5064587034; 2 Interdisciplinary Studies, School of Graduate Studies, University of New Brunswick, Fredericton, NB, Canada; 3 College of Community and Global Health, University of Manitoba, Winnipeg, MB, Canada; 4 George & Fay Yee Centre for Healthcare Innovation, University of Manitoba, Winnipeg, MB, Canada; 5 School of Health Sciences, UNSW Sydney, Sydney, New South Wales, Australia; 6 Falls, Balance and Injury Research Centre, Neuroscience Research Australia, Randwick, New South Wales, Australia

**Keywords:** acceptability, app, digital health, falls, balance

## Abstract

**Background:**

Falls are a major public health concern due to their impact on individuals and the health system. StandingTall (Ylva Health Pty Ltd) is a digital balance exercise app that has demonstrated strong adherence and fall-reduction effects in Australia. Before considering implementation in Canada, its acceptability must be assessed.

**Objective:**

This study aimed to explore the prospective acceptability of the StandingTall app among Canadian community-dwelling adults aged 50 years and older with varying levels of physical activity and technological skills.

**Methods:**

A concurrent mixed methods design was guided by the Theoretical Framework of Acceptability (TFA). After viewing a demonstration video, participants completed an online survey in which they rated the 7 TFA constructs using a 5-point Likert scale. Participants also reported demographic and health characteristics. A subsample completed interviews that were also based on TFA constructs. Convergence between data sources was assessed through overall patterns.

**Results:**

In total, 314 individuals completed the survey (mean age 61.8,  SD 8.0 y; n=279, 88.9% female participants) and 22 completed interviews. The mean acceptability score was 5.8  (SD 1.3; out of 7). Participants viewed the StandingTall app positively, valuing its convenience, flexibility, clear design, and alignment with their health and aging goals. Both data sources indicated strong acceptability, shaped by high affective attitude and self-efficacy and tempered by burden and opportunity cost factors related to technology issues and existing lifestyle commitments. Participants also reported mixed beliefs about its effectiveness, with some skepticism about the mechanisms by which the app’s exercises improve balance and ultimately reduce the risk of falls.

**Conclusions:**

Although the sample was heterogeneous, StandingTall was viewed as generally acceptable, with some heterogeneity across participants and with challenges that could be addressed using implementation strategies cocreated with potential users.

## Introduction

Falls among older adults continue to be a serious public health issue because of the high percentage of falls that result in emergency visits, injury-related hospital admissions, and deaths [1,2]. Falls adversely affect people in terms of their physical function, quality of life, loss of independence, hospitalization, morbidity, and mortality [3,4]. Robust evidence indicates that the most effective strategy to reduce falls among community-dwelling older adults is exercise that challenges balance, performed for 3 hours per week on an ongoing basis [5,6]. Furthermore, exercise also reduces the number of people experiencing fractures [6].

Community group exercise programs can help people access fall prevention exercise [7,8]. Some programs have been successful in reducing the risk of falls by improving physical performance (balance, mobility, and lower extremity strength) and by reducing the fear of falling [9]. However, many programs do not meet key criteria for effective fall prevention exercise (challenging balance and 3 h/wk on an ongoing basis); in Canada, for example, only 6% of community exercise programs do [10]. It has been suggested that adding technology-based options for participants to use independently to in-person offerings could lead to greater adherence to fall prevention guidelines among community-dwelling older people [11].

Mobile health has been defined by the Global Observatory for eHealth as medical and public health practice supported by mobile devices, such as mobile phones, personal digital assistants, patient-monitoring devices, and other wireless devices [12]. Mobile apps are software programs designed to run on smartphone or tablet platforms [13]. App-based programs can be ideal platforms for providing easy access to a target group and are cost-effective, mainly due to their accessibility and portability [14,15]. Importantly, technology-based interventions have been effective in reducing fall rates and improving adults’ balance and strength [16,17] High-quality apps and websites could supplement, and in some cases replace, in-person interventions [18], mainly because they can provide both exercise and tailored information and education [19]. Moreover, some apps can be used offline, which makes them accessible in rural areas or communities with limited internet access. In fact, as of 2024, only 62% of rural Canadians had access to high-speed internet [20]. This is also observed globally, where large portions of the world, particularly in Africa and Asia, face slow or nonexistent access [21].

The StandingTall app comprises balance exercises with embedded behavior change techniques, such as a weekly calendar for scheduling exercises, goal setting, and educational fact sheets [22]. Developed in Australia, StandingTall has demonstrated significant results in clinical trials, reducing injurious falls by 20% after 2 years [22]. This app does not require an internet connection, and participants aged 70 years and older have embraced it, reporting positive experiences, long-term benefits, and higher adherence rates than with other exercise interventions [22]. These outcomes suggest that the StandingTall app could serve as a complementary or sole option for performing balance-challenging exercises, especially in rural areas with limited internet access.

Acceptability is a concept that reflects the extent to which people delivering or receiving a health care intervention consider it to be appropriate, based on anticipated or experiential cognitive and emotional responses to the intervention [23]. Studying acceptability before implementation is recommended because even well-designed or effective programs are unlikely to succeed if the intended users are not willing or able to engage with them [23,24]. Understanding acceptability helps identify perceived barriers and facilitators, such as burden, usability, value alignment, and perceived effectiveness, so that interventions can be refined and adapted to meet user needs before large-scale rollout [25,26]. Examining acceptability also increases the likelihood of long-term adherence by ensuring that a program fits people’s values, expectations, and real-world contexts, which is particularly important for digital health interventions where engagement often declines over time [27,28]. Ultimately, assessing acceptability helps reduce implementation risks and may strengthen user-centered design in support of successful, scalable adoption of evidence-based interventions.

Although factors influencing the use of exercise apps among older adults remain unclear, it is reasonable to expect that baseline physical activity levels and digital literacy play important roles. According to the Capability, Opportunity, and Motivation model of behavior [29], the likelihood of adopting a new behavior or tool is influenced by an individual’s existing routines and perceived capabilities. For instance, individuals with lower baseline physical activity may perceive online exercise as a more accessible entry point, particularly if it reduces environmental barriers. Conversely, individuals who are already physically active may perceive a new digital intervention as difficult to integrate into their established routines, potentially reducing its adoption. Similarly, baseline digital literacy may shape app’s adoption: individuals with higher confidence and skills in using technology may be more likely to engage with exercise apps, whereas those with lower digital literacy may perceive greater barriers to use.

This study aimed to explore the prospective acceptability of the StandingTall app among Canadian community-dwelling adults aged 50 years and older with varying levels of physical activity and technological skills, using 2 data sources interpreted independently and concurrently.

## Methods

### Design Overview

This is a concurrent mixed methods study in which both quantitative and qualitative data were collected independently and approximately at the same time for a later simultaneous analysis. We integrated both data sources during analysis as proposed elsewhere [30,31]. The 2 data sources were developed based on the Theoretical Framework of Acceptability (TFA). The TFA was developed by Sekhon et al [23] through a systematic review and theoretical synthesis of how acceptability had been defined and measured across health care research. It comprises 7 constructs, as defined in Table 1. Subsequent widespread application across diverse health interventions [32] has provided strong conceptual and practical validity, demonstrating that the framework reliably captures the key dimensions influencing user acceptance. The TFA considers acceptability from 3 temporal perspectives: prospective (before participating in an intervention), concurrent (during the intervention), and retrospective (after participating in the intervention) [23]. In our case, the TFA was used prospectively, with the “thing” being the StandingTall app, and the acceptability was tested before participants had access while the app’s functions and purpose were explained.

**Table 1.** Constructs of the theoretical framework of acceptability and their application in this study.

Constructs	Definition	Our application
Affective attitude	How individuals feel about the “thing.”	Feelings about the StandingTall app (eg, interest).
Burden	Perceived amount of effort required to use the “thing.”	The perceived amount of effort required to use the StandingTall app (eg, downloading app).
Perceived effectiveness	Expectation that the “thing” will improve what is intended.	The perceived advantages of using the StandingTall app (eg, improve balance).
Ethicality	Fit between the “thing” and personal values.	The perceived fit of the StandingTall app with the individual’s value system (eg, lifestyle).
Intervention coherence	Understanding of how the “thing” works.	The extent to which the participants understand how the StandingTall app works (eg, features).
Opportunity cost	Benefits or activities that must be given up using the “thing.”	The extent to which other things need to be given up engaging with the StandingTall app (eg, other commitments).
Self-efficacy	Confidence to be able to use the “thing.”	The extent to which participants can perform the exercises within the StandingTall app (eg, exercise experience)

### Participant Recruitment

Participants were recruited through targeted Facebook (Meta Platforms, Inc) advertisements across Canada and through in-person outreach in public spaces such as community centers, libraries, and local events. Targeted Facebook advertisements were aligned with our inclusion criteria. Eligible participants were Canadians aged 50 years and older who self-identified as English speakers. Interested individuals received written information about the study and the informed consent form. Recruitment began in September 2024, and survey data collection concluded in January 2025.

Those who provided their consent to participate in the survey were directed to LimeSurvey, where the survey was hosted. From among the survey participants, we purposively recruited participants for the interviews via email.

### Data Collection

#### Survey

An online survey platform (LimeSurvey) was used to collect data. The survey lasted between 15 and 20 minutes, and participants watched a 5-minute video explaining the app, presenting testimonials, and answering questions about the app. The core of the survey asks questions related to the 7 constructs of the TFA using nine 5-point Likert-scale questions with verbal anchors (Multimedia Appendix 1). For example, a question related to self-efficacy was: “How confident are you in your ability to use this app?” Answers were strongly (5), moderately (4), indifferent (3), slightly (2), and not at all (1). Given the app’s balance-focused, fall-prevention nature, the perceived effectiveness construct was evaluated using 2 questions focused on exercise in general and one specifically on balance. Similarly, the self-efficacy construct was evaluated using 2 questions: one assessing confidence in using the app in general and the other assessing confidence in performing app-specific balance exercises.

The survey also asked demographic questions with multiple choice options, including an option to not answer. The questions asked about age, sex, gender, ethnicity, employment status, highest level of education, marital status, and household income. Participants also reported their chronic conditions (0 to 18) and daily prescribed medication. In addition, physical activity level was assessed using the Godin-Shepard Leisure-Time Physical Activity Questionnaire (GSLTPAQ) [33]. A score of 24 or more is considered active [33]. Finally, technology use was assessed via the FACETS (Functional Assessment of Current Employed Technology Scale) [34]. The FACETS provides a global sense of the participants’ general usage of common technologies. A score of 35 or more is considered a frequent or very frequent technology user [34]. At the end of the survey, participants could opt for an optional interview.

#### Interviews

Based on the survey, people with different levels of physical activity (above or below a score of 24 on the Godin questionnaire) and technology usage (above or below a score of 35 on the FACETS questionnaire) were invited to participate in the interviews. Interviews were conducted between December 2024 and March 2025. The original goal was to recruit 20 people as proposed for such a study design [35], analyze the data, and recruit more if needed. We stopped data collection when the data were sufficient and comprehensive enough to account for variations and identify recurrent patterns and when no substantial new insights were being generated [36].

The interviews were related to the app but independent of the survey responses. The interview guide was also aligned with the TFA (Multimedia Appendix 2). The initial app video was shown again, along with a second video on how to navigate and use the app. The interviews had 18 questions (2‐3 per construct) and took approximately 45 minutes. The interviews were conducted individually via Microsoft Teams and were recorded after obtaining consent. There were no prior relationships between the researchers and participants. These online interviews were conducted by a trained staff member who was not involved in data analysis.

### Data Analysis

#### Research Team and Reflexivity

Throughout the study, the research team used a post-positivist worldview [37,38] and engaged in reflexive practices by critically reflecting on their positionality, professional backgrounds, and assumptions to minimize potential bias. Reflexivity was maintained during data analysis and manuscript preparation to remain faithful to participants’ experiences. Data coding and analysis were conducted iteratively by MFFD and COTU, graduate students with backgrounds in aging and physical activity research. KD, KMS, and DRB were professors with expertise in active aging and fall prevention, and they designed and supervised all stages of the research process. KD is the founder, chief executive officer, and director of Ylva Health Pty Ltd, a company established to commercialize the StandingTall program. However, she was not involved in data analysis or in drafting the paper. None of the researchers was older than 50 years.

#### Surveys

Descriptive statistics for participant characteristics were reported as mean (SD) and n (%). Acceptability score was calculated as the mean (SD) scores for each TFA construct using the 5-point Likert scale. Construct scores were reported on a 5-point scale, except perceived effectiveness and self-efficacy, which were reported on a 10-point scale by summing scores from the 2 associated questions. Likert scale scores of acceptability were then dichotomized as 1 (yes) if participants indicated a 4 or 5 out of 5 levels of agreement, and 0 (no) if they indicated a neutral or lower level of agreement. For the questions assessing self-efficacy and perceived effectiveness, we used 1 (yes) if participants responded at least a 4-point level of agreement for both questions, and 0 (no) if participants indicated a 4-point level of agreement on only one question or anything lower than 4 on both questions. This dichotomization of acceptability was used as an indicator of consistently high acceptability across domains rather than as a primary measure of acceptability. All domains were weighted equally because the TFA conceptualizes acceptability as a multidimensional construct composed of distinct but complementary domains, and there is currently no empirical or theoretical guidance supporting differential weighting of individual constructs. The total acceptability score was calculated as the sum of the 7 questions (range 0‐7), and high acceptability was defined as a total score of 7 out of 7 and low acceptability as a total score of less than 7.

Independent *t* test and chi-square tests were used to compare demographic differences between those who reported a high vs a low acceptability. SPSS Statistics (version 30; IBM Corp) was used to conduct all statistical analyses.

#### Interviews

One research assistant transcribed the interviews conducted via Microsoft Teams into Microsoft Word. MFFD and COTU began the analysis by familiarizing themselves with the data through repeated reading of the transcripts. As a calibration exercise, MFFD and COTU independently coded one interview using a conventional content analysis approach [39] to generate initial codes directly from the data. They then discussed their coding with a third researcher (DRB) and reached consensus on an initial codebook consisting of 20 categories (Multimedia Appendix 3).

Using this coding framework, MFFD and COTU independently coded the remaining interviews and regularly compared their coding to ensure consistency throughout the process. When disagreements occurred, DRB reviewed the relevant excerpt and determined the most appropriate code. Once coding was completed and agreement was reached across all transcripts, MFFD and COTU conducted a deductive analysis, whereby the coded data were independently mapped to the 7 constructs of the TFA [23]. The same consensus process was followed to resolve discrepancies and finalize the categorization.

#### Data Integration

Quantitative and qualitative data were integrated to understand how participants’ perceptions and contextual circumstances shape the prospective acceptability of the app. For each TFA construct, survey results and interview findings were examined for points of convergence and divergence. Findings were considered similar (convergent) when both data sources showed the same directional pattern, for example, high survey ratings accompanied by predominantly positive interview accounts, or low ratings accompanied by consistent concerns. Findings were considered different (divergent) when survey results and interview data reflected conflicting patterns, for example, constructs with high quantitative ratings alongside predominantly skeptical qualitative narratives. We present the quantitative and qualitative findings together to support an integrated interpretation of acceptability across domains, explaining how participants’ perceptions and contextual circumstances shape the acceptability constructs.

### Ethical Considerations

The Research Ethics Board of the University of New Brunswick reviewed and approved this study, which is on file as REB 2024‐126. Participants of this study provided written informed consent before answering the survey. They had the option to discontinue their participation at any time. Participants recruited for interviews were informed that the interviews were being recorded and provided an additional verbal consent to continue their participation. All survey responses and interview data were stored on secure servers at the University of New Brunswick and were identified only by study IDs. Interview transcripts were deidentified before analysis, and the results were reported in aggregate. Participants who completed the interview received an incentive of CAD $25 e-gift card (CAD $1=US $0.72 as of August 10, 2026).

## Results

### Participant characteristics

A total of 314 individuals completed the survey. The average age was 61.8 (SD 8.0) years, and they were predominantly White (n=298, 94.9%) and female participants (n=279, 88.9%). On average, the participants had a few chronic conditions (1.3, SD 1.4). Participants were generally physically active, with an average score of 27.4 (SD 22.0). However, most were not involved in any exercise programs, either in-person or online, with 28.3% (n=89) participating in one. Furthermore, 19.4% (n=61) of the total participants specifically engaged in an online exercise program. Among those who reported participating in any exercise program, 22.3% (n=70) indicated that their program specifically targeted fall prevention. Overall, participants had high technology usage (mean FACETS score 36.9, SD 7.3). Most participants owned a technological device such as a desktop computer, laptop, smartphone, or tablet. Most participants reported owning a smartphone (n=294, 93.6%), a laptop (n=244, 77.7%), or a tablet (n=215, 68.5%), with desktop computers being the least common (n=109, 34.7).

A total of 22 participants were interviewed. The average age was 63.9 (SD 8.9) years, and they were predominantly White (n=21, 95.5%) and female participants (n=17, 77.3%). This subsample of participants had fewer chronic conditions (mean 1.0, SD 1.3) than individuals participating in the survey only. Participants were more physically active (mean GSLTPAQ score 37.3, SD 26.9) but had lower technology use, with an average FACETS score of 32.7 (SD 9.1; Table 2).

**Table 2.** Demographic characteristics of participants.

Characteristics	Participants (n=314)	Interviews (n=22)	High acceptability (7/7, n=121)	Low acceptability (<7/7, n=193)
Age (y), mean (SD)	61.8 (8.0)	63.9 (8.9)	61.9 (7.8)	61.7 (8.2)
Sex: female, n (%)	279 (88.9)	17 (77.3)	113 (93.4)	166 (86.0)
Gender: woman, n (%)	275 (87.6)	17 (77.3)	112 (92.6)	163 (84.5)
Ethnicity: White, n (%)	298 (94.9)	21 (95.5)	117 (96.7)	181 (93.8)
Occupation: retired, n (%)	150 (47.8)	14 (63.6)	64 (52.9)	86 (44.6)
Education: university degree, n (%)	175 (55.7)	16 (72.7)	67 (55.4)	108 (55.9)
Marital status: married, n (%)	205 (65.3)	15 (68.2)	82 (67.8)	123 (63.7)
Household income (>CAD $100,000 per year), n (%)	100 (31.8)	5 (22.7)	29 (23.9)	71 (36.8)
Sum of chronic conditions (out of 18), mean (SD)	1.3 (1.4)	1.0 (1.3)^a^	1.3 (1.5)	1.2 (1.4)
Number of medications taken per week, mean (SD)	2.3 (1.9)	2.7 (2.0)	2.6 (2.0)	2.2 (1.9)
Computer ownership: yes, n (%)	109 (34.7)	7 (31.8)	39 (32.2)	70 (36.3)
Laptop ownership: yes, n (%)	244 (77.7)	14 (63.6)	91 (75.2)	153 (79.3)
Smartphone ownership: yes, n (%)	294 (93.6)	21 (95.5)	117 (96.7)	177 (91.7)
Tablet ownership: yes, n (%)	215 (68.5)	13 (59.1)	86 (71.1)	129 (66.8)
GSLTPAQ score (≥24: active), mean (SD)	27.4 (22.0)	37.3 (26.9)^a^	25.2 (20.7)	27.9 (21.9)^b^
FACETS^c^ score (≥35: frequent use of technology), mean (SD)	36.9 (7.3)	32.7 (9.1)^a^	38.5 (5.7)	35.9 (7.9)

a *P*<.05 between participants who only completed the survey and those who completed the interview.

b *P*<.05 between high and low acceptability.

c FACETS: Functional Assessment of Current Employed Technology Scale.

### Quantitative Findings

The mean acceptability score was 5.8 (SD 1.3; out of 7); the highest-rated construct was self-efficacy, with an average score of 9.3 (SD 1.2; out of 10), followed by affective attitude (4.5, SD 0.6; out of 5) and intervention coherence (4.4, SD 0.8; out of 5). The lowest-rated construct was burden, with an average of 3.7 (SD 1.3; out of 5), followed by ethicality (3.9, SD 0.9; out of 5). A total of 38.5% (121/314) of participants reported high acceptability, rating all questions as at least 4 (moderately) or 5 (strongly). Affective attitude (296/314, 94.3%), self-efficacy (294/314, 93.6%), and intervention coherence (292/314, 92.9%) were the constructs with the highest proportions of participants indicating an acceptability level of at least 4 out of 5. Burden (196/314, 62.4%) and ethicality (226/314, 71.9%) were the constructs with the lowest proportion of participants expressing an acceptability level of at least 4 out of 5.

### Qualitative Findings

All the 7 TFA components were represented in the interviews. Pertinent to affective attitude, participants expressed a positive attitude toward StandingTall, mainly due to its perceived convenience, ease of use, and flexibility: *“*I don’t see any downside to it actually” (P12)*,* and “Just from the video it looks like it would be really helpful to like show you the exercise” (P18). Regarding ethicality, many felt that the app aligned with their health goals, “I do wish to make that change. I don’t like being afraid of walking on snow” (P12), and values towards a healthy and active aging, “I’m aging well. I don’t have any major health issues and I’m trying to keep it that way, but I know things can change” (P19).

Regarding intervention coherence, participants expressed strong confidence in their understanding and potential to use the app. For instance, P7 expressed: “What I liked about the idea of StandingTall is that it’s individualized. And as you progress, they get you to do activities to progress more. I like that idea.” This perception was strengthened by the app’s individualized and adaptable programming, which allowed them to choose their session length and progression at their own pace:

*I like that it’s very tailored to the person doing it. I think that’s really good. You can also choose how long you do different exercises for which so you can prioritize things for yourself.*

[P4]

Burden and opportunity cost were related to technology ownership: “Well, my only challenge is that I don’t have an iPad, so it’s really small on my phone and it sounded like it wouldn’t work on a MacBook.” (P4)*,* and to contextual barriers such as internet connection: “I guess maybe connectivity like that would be one issue. You know dropping signal” (P1). Furthermore, some participants expressed no challenges in including the app in their routine, but sometimes their active and busy lifestyles seemed to interfere with the app use:

*It depends on how many times I get called for bowling, that can be up to twice a week. And then there’s line dancing and the exercise class twice a week.*

[P6]

Perceived effectiveness was moderately high but accompanied by some uncertainty regarding the app’s underlying mechanisms as expressed by P20: “I think balance is a difficult thing to do, I don’t understand how you can do that exercise and increase your balance.” Finally, participants’ confidence in their ability to engage with the app seemed to vary depending on their perceived need for supervision and their ability to perform the exercises safely as expressed by: “I would be more comfortable having somebody there (while exercising)” (P9), and “I’m not too concerned about injuring myself when I’m doing the exercises” (P13).

### Integrated Acceptability Findings: by TFA Construct

**Figure 1.**
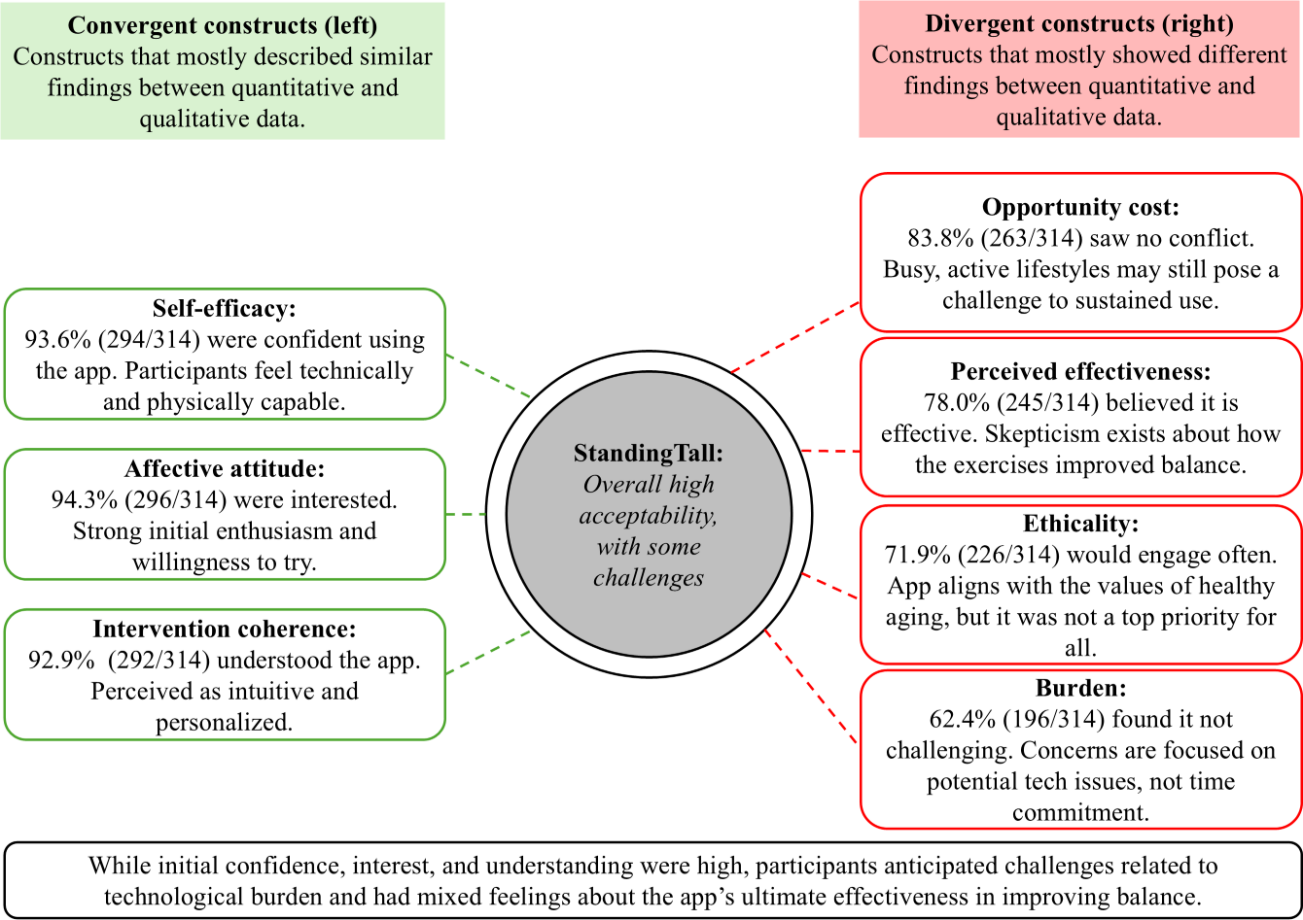
Summary of findings with convergent and divergent acceptability constructs based on data integration.

Figure 1 summarizes the findings and compares constructs that were similar or different between the quantitative and qualitative data.

#### Affective Attitude

Overall, findings from both data sources indicate a highly positive affective attitude toward the app. A total of 296 (94.3%) participants reported being at least somewhat interested in the app. This strong level of interest was echoed in the interviews, where participants expressed willingness and motivation to engage with the app. As one participant stated, “I would definitely try anything, at least to give it a try and see if it’s something I would like to do” (P11). Although some participants indicated that they would prefer to use it to have a more accurate and informed opinion on it, it was perceived as appealing and user-friendly in principle: “I would like to use it and take a look at it and work with it as opposed to just seeing it in motion” (P8). Together, these findings demonstrated strong affective acceptability, characterized by high interest, openness, and initial enthusiasm toward the app.

#### Burden

Burden emerged as the lowest-rated construct in the survey, with only 62.4% (196/314) of participants indicating that incorporating the app into their routine was slightly challenging or not challenging at all. Interview findings helped explain this pattern. Overall, participants did not express that it would require a lot of effort to use the app, especially when used in short bouts:

*I don’t think initially incorporating it should be difficult. Like you know, 20 minutes a day or even doing it in, you know, smaller bites sometimes and more on another day, like that should be manageable.*

[P15]

However, participants anticipated some difficulties, most notably related to technological issues. As one participant expressed: “Technology is great as long as it’s working. When it doesn’t work, that’s when I lose it. But I don’t have a whole lot of tricks in my bag for what to do when things don’t work” (P22). The integration of these findings suggests that while the practical demands of the app were perceived as manageable, concerns regarding technology and troubleshooting may contribute to the moderate burden ratings observed in the survey.

#### Ethicality

Survey findings indicated a moderate to high level of alignment between the app and the individuals’ value system, with only 71.9% (226/314) of participants reporting that they would engage with the app often or very often. Interview data provided insight into the values and beliefs underlying this intention to engage with the app. Participants frequently described strong alignment between the app and their personal values, particularly regarding the importance of exercise, functional independence, and healthy aging. For instance, participants explained: “I do believe in exercise, I guess, and things, and you know, keeping functional abilities” (P13)*;* “Healthy aging and adapting activities to changing abilities is an important idea to me” (P16). In addition, participants perceived that the app would contribute to their overall health and wellness goals, and it was seen as a useful tool for maintaining physical function and preventing decline with aging:

*I have to heal, and the new life I’m hoping to get includes a regular exercise routine of some kind, a safe one like the one that’s proposed in this app.*

[P19]

*That’s my attitude; you got to keep moving. I don’t sit a lot. I don’t want my bones to start falling apart so that’s my biggest concern right now. I mean, I’ve weighed the same all my life. I haven’t gained, it doesn’t matter what I eat or don’t eat. I’m quite healthy, so I just don’t want my bones to disintegrate.*

[P17]

#### Intervention Coherence

Overall, participants expressed a strong understanding of the app and its intended function and goals. Quantitatively, 92.9% (292/314) of participants indicated a moderate to strong understanding of how the app works and what it looks like. This was supported by interview findings, in which participants described the interface as intuitive and easy to navigate: “I thought it looked pretty clear. It seemed to guide people through the screens pretty easily” (P4). Participants also expressed particular appreciation for the assessment and feedback features of the app, especially for helping them situate themselves at the appropriate exercise level and monitor their progress:

*I’d like to have a different lens on what I’m doing, and maybe some different types of exercise to sort of keep the balance and then have that progress and make the exercises more difficult as you progress - that interests me.*

[P15]

The fact that the intensity level progresses as they use the app and are reassessed reinforces their confidence in the app:

*I’m thinking that, you know, once it sees you do it, and enters whatever your information. That puts you in a you know, if you’re doing really well, then it moves you up a little bit harder. And so that’s good.*

[P11]

Participants also described the app as more personalized to one’s individual needs and positioned the app as advantageous over other existing exercising tools:

*I think the idea that it would be looking at where you’re at and then making it progress is to be harder to meet your specific needs, really is very beneficial and something that we don’t always get as well with other programs.*

[P5]

The integration of these findings indicated strong intervention coherence among participants, characterized by a clear understanding of the app’s functionality.

#### Perceived Effectiveness

The survey results suggested generally positive expectations regarding the app’s effectiveness, with 78.0% (245/314) of participants rating it as likely to be effective in improving balance and preventing falls. However, interview data revealed divided opinions on perceived effectiveness. Some participants expressed that the app would help keep them physically active and viewed it as a preventive exercise tool: “I think that would be really helpful, and preventative as we go as we age into our older years” (P3). Conversely, others expressed skepticism about whether the exercises would help to meaningfully improve balance and prevent falls. As one participant stated, “I don’t understand how you can do those exercises and increase your balance. I think balance is important, but I’m not sure how you can instill it in people.[...] I would have to say, I don’t know that I believe that they can make a lot of difference or make an odder difference for me” (P20). Furthermore, other participants reported a lack of understanding of why the app could be effective as it is: “They look like, I mean, I don’t really have a good concept of what balance improvement exercises would be, but those looked like nothing was odd or surprising about them” (P18).

Despite these reservations, participants also indicated that the app could help overcome some barriers associated with in-person exercise programs, such as weather and mobility constraints:

*I do think that it’s a great idea, especially, you know, for a lot of older adults like in the winter that they don’t want to go out necessarily, even if they do it, go out to some kind of exercise like it gives an option that you don’t have to go out on an icy day, you can just do your thing at home.*

[P15]

Overall, the integrated findings suggest moderately high perceived effectiveness, tempered by differences in participants’ confidence regarding the app’s specific mechanisms of impact.

#### Opportunity Cost

A total of 83.8% (263/314) of participants reported that the app was unlikely to conflict with other activities. Nevertheless, interview data indicated that existing commitments and lifestyles, including participation in different recreational activities and other organized sports or programs, shaped individuals’ responses and commitment to the app. For instance, participants stated that their involvement in other recreational activities could limit their capacity to incorporate the app into their routine:

*Well, we swim, and I garden. I have vegetable gardens and a few flower gardens… I bike, we hike, we camp, that kind of thing. We’re outdoors as much as we can be.*

[P22]

They also perceived that others would have similar conflicts:

*My neighbors on the other side go to the Y, go to yoga, play rock pickleball. I don’t know if they’d have time to put it in their schedule.*

[P12]

Previous history of barriers to exercise, such as personal and familial responsibilities, was also seen as a potential challenge to using the app:

*I have a pretty complicated family life with one of my kids in particular that makes it a bit hard for me to get out and do exercise, but I’m just. I just feel so tired, and then whenever I do start to exercise, I find that even if it’s in a guided context, I have a tendency to strain something or injure something.*

[P14]

Thus, although opportunity cost was rated favorably overall in the survey data, the qualitative findings suggest important contextual constraints that may affect sustained participation in the app.

#### Self-Efficacy

In relation to self-efficacy, 93.6% (294/314) of participants indicated that they were moderately to strongly confident in their ability to use the app. Moreover, 94.3% (296/314) of participants felt confident in their ability to perform the exercises recommended by the app. Interview data illustrated several factors that supported participants’ confidence, including previous exercise experience: “I can do this at the stand, and I do the one-leg standing, and I do something with yoga moves, but I’m not as consistent with that piece, and I know that it’s an important part, but it’s hard to just work everything in” (P14), technology literacy, “I am comfortable with technology” (P12)*,* and previous experience with digital exercise formats: “During the pandemic, we did go online, and I did yoga online with them. So I’m experienced doing exercises online” (P12).

Overall, participants expressed that the exercises seemed appropriate, safe, and manageable. A participant stated, “Those [exercises] are perfectly, perfectly reasonable. And you know, not that hard to do. I mean, I might need a chair to have a chair beside me till I get better at it, but yeah” (P7)*,* and this was further supported by, “I’m not so concerned about the safety because I feel like it’s I’m not the stage where I’m likely to fall over and break something if I turn the wrong way. That definitely would be useful, but I feel like I can probably fairly accurately copy, you know, somebody on screen” (P10). The integration of these findings indicates strong self-efficacy, with participants expressing both behavioral and technological confidence in engaging with the app.

## Discussion

### Principal Findings

This study aimed to explore the prospective acceptability of the StandingTall app among Canadians aged 50 years and older living in the community. Despite a recruitment bias leading to a sample of mostly White women who are active and are fairly frequent technology users, findings demonstrate strong prospective acceptability of the StandingTall app among older adults. Participants showed positive attitudes, high confidence, and a strong understanding of the app’s purpose. They valued its convenience, personalization, and potential to support healthy aging. However, contextual factors such as technology challenges, varying beliefs about balance training, and busy lifestyles moderately reduced perceived burden and opportunity cost scores.

Notably, only 38.5% (121/314) of participants reported high acceptability across all constructs, suggesting that while the app was broadly well-received, acceptability was not uniformly high. This variability emphasizes the importance of tailoring implementation strategies to address individual differences in ability, preferences, and contextual constraints. These findings indicate that future implementation of the app should prioritize reducing perceived burden and strengthening value alignment between the app and the users. In practice, this means clearly linking the app’s features to outcomes that older adults consider meaningful, such as maintaining independence, improving balance and mobility, preventing falls, or managing chronic conditions. Emphasizing these benefits during onboarding, providing feedback that shows how app use supports these goals, and framing the app as a tool to support healthy aging may help increase its perceived relevance. Especially for older adults, it has been reported that adherence to mobile health greatly depends on their motivation and support received, including training and resources [40,41]. Both data sources highlighted that while prospective initial acceptability might be strong, the conditions highlighted above–value alignment and perceived relevance–would be key in long-term usage.

The utility of the concept of intervention acceptability lies in its ability to predict key outcomes of interest, such as user engagement, intervention effectiveness, and widespread adoption at the local, national, and international levels [27]. As acceptability is influenced by prevailing social and cultural norms, which are deeply contextual and ever-changing [27], this study enabled us to understand some factors specific to the Canadian population, guided by the TFA.

Our findings suggest convergent evidence of an affective attitude toward the app, characterized by strong interest, openness, and initial enthusiasm. This could be partially explained by the sample recruitment strategies that included online recruitment of technology literate and physically active individuals. However, this initial enthusiasm could be overtaken by other factors, such as burden or a lack of understanding of the effectiveness mechanisms, leading to decreased long-term engagement. This is important to consider because, as the intervention develops through pilot, feasibility, efficacy and implementation trials, the purpose of assessing acceptability may change from ensuring it is not unacceptable to predicting future use of or satisfaction with the intervention. If perceived appropriateness is not found in the later stages, the intervention may not be unacceptable, but it may not be used [42].

The burden was not necessarily the time required to use the app, but rather the stress associated with technology issues. Previous research has reported that apps that keep crashing or are poorly built are rated very poorly and are therefore used less frequently [27]. Thus, addressing technical issues may decrease perceived burden and reduce withdrawal rates. Furthermore, even though the StandingTall app does not require an internet connection to work, implementation efforts should account for the initial need for an internet connection so individuals can access and use the app adequately. This is of particular importance in contexts like Canada, where environmental resources, context, and geographic vastness can influence implementation efforts [43].

One of the most positive findings was the participants’ high appreciation for the app’s ability to monitor progress. Participants noted that the continuous assessment of physical function and the ability to increase the difficulty of the exercises made it more professional and personalized than other existing tools. This was valuable for participants and could act as a key driver of confidence and sustained interest, especially as tailored screening and user-centered approaches have demonstrated to increase usability and perceived value [44]. Moreover, smartphone-based features for assessment, tracking, and treatment have been helpful in improving mental health outcomes [15].

A significant nuance existed in how participants viewed their time. While 83.8% (263/314) of participants reported that the app would not conflict with other activities, interview data showed a highly active, busy lifestyle that could realistically limit sustained participation, a common barrier to physical activity adherence in adults living in the community [45]. This could signal a risk that the app may be viewed as redundant or less appealing than other social, recreational, or outdoor activities. However, the app was also seen as a valuable option for the winter, to overcome barriers related to inclement weather and mobility constraints when outdoor activities are not available.

Regarding the app’s effectiveness in improving balance and reducing the risk of falls, there was a notable discrepancy between quantitative survey results and qualitative interview insights. While 78.0% (245/314) of participants expected the app to be effective, interviews revealed skepticism about the nature of the exercises and mechanisms through which they would improve balance. Some participants perceived the exercises as very simple and ineffective at meaningfully improving balance or preventing falls, suggesting a misunderstanding of balance training and its importance. Similar results have been reported previously in falls prevention, specifically in perturbance balance training among older adults, where recognizing training effects was not clear even after receiving the intervention [46]. In the context of acceptability, this calls for better education not only on the app to explain the scientific rationale of the specific content and its benefits, to increase participants’ understanding of the exercises they would perform, but on the components of balance training and practical applications in real life. Additionally, participants may question if it is at all possible to prevent a fall in daily life, as the belief that falls are accidental and inevitable is well known from the literature [47]. Therefore, increasing knowledge can be an important facilitator of physical activity [48], and this may be translated into digital interventions as well.

This study distinguished 2 key components of participants’ confidence in using the app: physical literacy (their ability to perform the exercises) and technology literacy (their ability to use technology efficiently), both of which were high among the study population. These physical and psychological capability factors have been reported as necessary for adoption and adherence to physical activity and exercise [48]. Moreover, this high usability and ease of use have been reported as critical for the implementation and adoption of apps [44]. However, this physical activity and technological bias suggests a potential gap with those with less access to physical activity opportunities and limited experience exercising, as well as those with less technical skills to use technology.

Overall, the characteristics of the StandingTall app make it strongly acceptable among Canadians, as it aligns with previous research that showed that increased acceptability and user satisfaction were associated with interventions that were seen as time and cost-efficient, requiring the acquisition of minimal resources or no new skills, which used coherent language, and provided tailored information to support the individuals and their autonomy [49,50]. This is encouraging as only 28.3% (89/314) of participants were currently engaging in exercise programs, which might change with the use of the app that they found acceptable.

### Strengths and Limitations

The primary strength of this study is the use of a well-established theoretical framework, TFA, and a mixed methods design to evaluate the prospective acceptability of the StandingTall app. Using the TFA not only allowed us to determine that the app was acceptable but also to identify specific factors across the 7 constructs, highlighting the app’s strengths and weaknesses. Furthermore, the mixed methods approach helped reveal contradictions between participants’ optimistic survey responses and their practical realities, thereby enabling a better understanding of the app’s real-world acceptability. By integrating both data sources, this study moved beyond prospective initial enthusiasm to identify contextual factors necessary for long-term success, which might help shape strategies to improve acceptability and engagement once the app is introduced in Canada.

The prospective acceptability measured in this study means that participants evaluate the app based on its concept and a short demo, rather than through sustained practical use. Some participants stated that they would need to use the app over time to have a more accurate and informed opinion. Consequently, the high initial positive attitude might not necessarily translate to long-term adherence. However, other studies have reported that prospective and experienced acceptability were significantly positively correlated, indicating that, in some cases, the acceptability of an intervention can be adequately gauged from an anticipated acceptability study prior to an expensive pilot or feasibility study [42].

Another limitation is that the dichotomization of acceptability may have reduced the sensitivity to differences between participants, and it needs to be interpreted in addition to the continuous scores for better integration of results.

We acknowledge that the use of convenience sampling and Facebook-based recruitment likely introduced selection bias. Participants who responded to our online advertisements may be more comfortable using technology, have greater internet access, and be more interested in digital health interventions than the broader population of adults aged 50 years and older. As a result, the sample may not fully represent the target population, limiting the generalizability of the findings. Due to the characteristics of our sample, findings may not reflect the perspectives of men and adults with limited digital literacy, lower technology usage, or restricted internet access. However, it is quite possible that technology-based interventions are more likely to attract people who are somewhat active and technology literate. People who are completely inactive and/or not familiar with technology might need to increase their readiness before adopting a technology to become more physically active. From an implementation perspective, studying individuals who are already interested in digital health interventions and are comfortable with technology provides valuable information regarding factors that facilitate uptake among potential users.

These findings can inform strategies to support initial adoption before expanding research to populations that may be more resistant to technology or currently physically inactive. Overall, future studies should use more diverse recruitment strategies to test acceptability in diverse communities.

### Conclusions

Despite a recruitment bias leading to a sample of mostly White women who are physically active and fairly frequent technology users, this study provides evidence that the StandingTall app is generally favorable but heterogeneous among Canadian community-dwelling adults aged 50 years and older. By integrating the TFA into a mixed methods approach, this study provides a robust foundation for assessing the app’s acceptability among Canadians. Future research should extend this work by examining acceptability in relation to implementation considerations and among diverse samples.

## Supplementary material

10.2196/95812 Multimedia Appendix 1 Survey questionnaire used to assess participant characteristics, physical activity, technology literacy, and prospective acceptability of the StandingTall app.

10.2196/95812 Multimedia Appendix 2 Semistructured interview guide used to explore the prospective acceptability of the StandingTall app among community-dwelling Canadians aged 50 years and older.

10.2196/95812 Multimedia Appendix 3 Preliminary and final coding framework used for the qualitative analysis.

## Abbreviations

FACETS: Functional Assessment of Current Employed Technology Scale
TFA: Theoretical Framework of Acceptability
