# Supplementary material for: Understanding the Prospective Acceptability of a Fall Prevention Exercise App Among Canadians Aged 50 Years and Older Living in the Community: Mixed Methods Study
Source: JMIR Form Res. 2026 Aug 13;10:e95812. doi: 10.2196/95812 (PMC13472487; doi:10.2196/95812)
Supplement: Supplementary file 1 [file formative-v10-e95812-s001.docx]

**Appendix 1**

**Survey**

[VIDEO](https://www.veed.io/view/2159c166-c58c-4bd3-8e28-27dcf87f74d2?panel=share)

A. **DEMOGRAPHIC**
1. Current Age:
2. Sex: Male/Female
3. Gender
   - Male
   - Female
   - Nonbinary
   - Genderfluid
   - Transgender
   - Other:
   - I prefer not to say
4. Marital Status:
   - Single
   - Married
   - Other
5. Employment status
   - Full-time employment
   - Part-time employment
   - Retired
   - Other
6. Which ethnic group or cultural group do you identify with? Check all that apply

- White
- Arab
- Black
- Chinese
- Filipino
- Indigenous
- Indo-Caribbean, Indo-African, Indo-Fijian
- Japanese
- Korean
- Latino
- North African (e.g., Egyptian, Libyan)
- Oceania
- South Asian (e.g., Bangladeshi, Pakistani, Indian, Sri Lankan, Punjabi)
- Southeast Asian (e.g., Cambodian, Malaysian, Thai, Vietnamese)
- West Asian & Middle East (e.g., Afghani, Armenian, Iranian, Iraqi, Israeli, Jordanian, Lebanese, Palestinian, Syrian, Yemeni)
- Other (Please specify):
- I prefer not to answer.

7. Please indicate your highest achieved level of education.

- No certificate, diploma or degree
- High school diploma or equivalency certificate
- College, CEGEP or other non-university certificate or diploma
- University certificate or diploma below bachelor level
- Prefer not to answer

8. Household income:

- <50,000
- 50,000-100,000
- 100,000 and more
- Prefer not to answer.

**B. PHYSICAL ACTIVITY LEVEL: GODIN LEISURE-TIME EXERCISE QUESTIONNAIRE**

During a typical 7-Day period (a week), how many times on the average do you do the following kinds of exercise for more than 15 minutes during your free time (write on each line the appropriate number).

A) Strenuous Exercise (heart beats rapidly) (e.g., running, jogging, hockey, football, soccer, squash, basketball, cross country skiing, judo, roller skating, vigorous swimming, vigorous long-distance bicycling).

Times per week____

B) MODERATE EXERCISE (NOT EXHAUSTING) (e.g. fast walking, baseball, tennis, easy bicyling, volleyball, badminton, easy swimming, alpine skiing, popular and folk dancing)

Times per week____

C) MILD/LIGHT EXERCISE (MINIMAL EFFORT) (e.g. yoga, archery, fishing from the river banks, bowling, horseshoes, golf, snow-mobiling, easy walking)

Times per week____

9. Do you regularly participate in an exercise program in your community or online?

- Yes
- No

10. Do you regularly participate in an exercise program online?

- Yes
- No

11. If you answered yes at question 9 or 10, do any of these programs aiming to reduce falls?

- Yes
- No

**C. TECHNOLOGY LITERACY**

Functional Assessment of Currently Employed Technology Scale (FACETS) Questionnaire

12. I send emails

- Never
- A few times a year
- A few times a month
- Once a week
- A few times a week
- Daily

13. I find, open and close files on my computer

- Never
- A few times a year
- A few times a month
- Once a week
- A few times a week
- Daily

14. I send text messages using a smart phone

- Never
- A few times a year
- A few times a month
- Once a week
- A few times a week
- Daily

15. I post on social media (e.g. facebook)

- Never
- A few times a year
- A few times a month
- Once a week
- A few times a week
- Daily

16. I manage my banking and credit card accounts online...

- Never
- Tried but it didn't work
- Got help but didn't work
- Only with help
- Can but prefer not to
- Prefer to

17. I pay bills and make purchases via the internet…

- Never
- Tried but it didn't work
- Got help but didn't work
- Only with help
- Can but prefer not to
- Prefer to

18. I communicate with my doctor or clinic online…

- Never
- Tried but it didn't work
- Got help but didn't work
- Only with help
- Can but prefer not to
- Prefer to

19. I communicate with my health insurance company online…

- Never
- Tried but it didn't work
- Got help but didn't work
- Only with help
- Can but prefer not to
- Prefer to

20. I have installed components (monitors, speakers, mice) …

- Never
- Tried but it didn't work
- Got help but didn't work
- Only with help
- Can but prefer not to
- Prefer to

21. I have reset a modem or router in my home…

- Never
- Tried but it didn't work
- Got help but didn't work
- Only with help
- Can but prefer not to
- Prefer to

22. Select the electronic device(s) you own from the list below.

- Computer (screen + box)
- Laptop
- Smartphone
- Tablet

23. Do you have access to reliable internet at home?

- Yes
- No

If you answered no in the previous question, can you access reliable internet anywhere (e.g., a friend/family home, library, church, community centre)?

- Yes
- No

WATCH THE DEMO

24. What are your thoughts on the video provided to you? Please select the statement that best applies to you. The video was extremely informative and provided me with extended knowledge on the app

- The video was informative and provided me with knowledge on the app
- The video was somewhat informative and allowed for some knowledge on the app
- The video allows me to gain for information and knowledge but I still feel I don’t understand the app or have several questions
- I did not find the video informative or I did not gain any knowledge on the app

25. Based on your introduction to the Standing Tall app, how interested are you in trying the app?

- Very interested
- Somewhat interested
- Indifferent
- Not interested unless I get more details
- Not interested at all

26. How challenging do you predict it would be to incorporate this app into your weekly routine?

- Extremely Challenging
- Moderately challenging
- Indifferent
- Slightly challenging
- Not challenging

27. How much does this app relate to your exercise goals (e.g., more fit, more functional, weight loss)?

- Strongly
- Moderately
- Indifferent
- Slightly
- Not at all

28. What is your level of understanding of the app? (e.g. how it works, what is required, what is offered)

- Strong understanding
- Moderate understanding
- Indifferent
- Slight understanding
- No understanding

29. How likely is this app to conflict with your prior engagements ?

- Very Likely
- Somewhat likely
- Likely
- Somewhat unlikely
- Not likely

30. Do you think that by completing the exercise provided in the app you can improve your balance?

- Very Likely
- Somewhat likely
- Likely
- Somewhat unlikely
- Not likely

31. How confident are you in your ability to use this app?

- Strongly
- Moderately
- Indifferent
- Slightly
- Not at all

32. How confident are you that you will be able to do the app’s recommended exercises?

- Strongly
- Moderately
- Indifferent
- Slightly
- Not at all

33. How often would you engage with this app?

- Very Often
- Often
- Somewhat often
- Not often
- Not at all

**D. MEDICATIONS AND CHRONIC CONDITIONS**

34. How many prescribed medications do you take every week?

- 0-1
- 2-3
- 4-5
- 6+

35. Do you currently have any of the chronic conditions listed below?

A) Arthritis

- Yes
- No

B) Edema

- Yes
- No

C) Asthma

- Yes
- No

D) Emphysema

- Yes
- No

E) Blood pressure

- Yes
- No

F) Cardiac or circulation problems

- Yes
- No

G) Diabetes

- Yes
- No

H) Stomach ulcer

- Yes
- No

I) Digestive chronic conditions

- Yes
- No

J) Liver disease

- Yes
- No

L) Urinary problem

- Yes
- No

M) Osteoporosis

- Yes
- No

N) Anemia

- Yes
- No

O) Thrombosis

- Yes
- No

P) Stroke

- Yes
- No

Q) Parkinson`s disease

- Yes
- No

R) Muscular dystrophy

- Yes
- No

S) Thyroid problem

- Yes
- No

Thank you for taking the time to complete this survey. You may share your thoughts by completing a 30 minute interview online, over the phone or in person. If you are interested in participating in the interview, please indicate how you would like to complete it and how you would like to be contacted. Please note that you may choose to complete only the survey.

Are you interested in completing the interview

- Yes
- No

If yes, please indicate your preference and contact information.

- Online
- Over the phone
- In-person
