# Supplementary material for: Understanding the Prospective Acceptability of a Fall Prevention Exercise App Among Canadians Aged 50 Years and Older Living in the Community: Mixed Methods Study
Source: JMIR Form Res. 2026 Aug 13;10:e95812. doi: 10.2196/95812 (PMC13472487; doi:10.2196/95812)
Supplement: Supplementary file 2 [file formative-v10-e95812-s002.docx]

**Appendix 2**

**Interview guide**

Thank you for participating in this important study to understand your interest in using this new app to exercise in the comfort of your home as an addition to what you currently do or as a sole strategy to become more active and reduce your risk of falling. After watching the video demonstration, you shared your initial thoughts on the app in the survey. We will now discuss your feelings about using applications, particularly the Standing Tall app.

Before we start, I want to remind you that your responses are confidential, and you can withdraw from the study without any consequences. Do you have any questions before we begin?

This study examines the Standing Tall exercise app. Participants will be asked questions related to their preferences and interests.

1. Are you currently using smartphone or tablet apps, and for what purpose?
2. Do you routinely exercise?
A. If yes, how much, when and where do you do your exercises?
B. If not, what are your main barriers?
3. Are you currently engaged in an exercise program offered in your community, or have you been in the past? If so, tell me about it.

A. If yes, was this program a community-based fall prevention exercise program?

4. How do you feel about the idea of exercising in front of a device?

5. What are your thoughts on using an app to access an exercise program?

As mentioned in the documents you read before filling out the survey, an app called Standing Tall was developed for people age 70+ to use on their own time to be active and get benefits. Let's start by showing you the promotion video again.

SHOW VIDEO

1. What questions would you have before engaging with the app?
2. What about this app interests you?
3. How do you feel about navigating the app?
4. What challenges do you anticipate by completing the exercise provided in the app?
5. What challenges do you anticipate could arise by including the app in your weekly routine?
6. Do you anticipate reasons for not using this app (e.g., chronic pain/illness, insufficient physical space to complete exercises, time, technology)?
7. How does this app help you achieve your health goals?
8. Based on the video and exercise provided, what do you think this app seeks to accomplish?
9. Based on the video, do you think this exercise program can improve your balance and reduces your risk of falls?
10. What are your thoughts on the exercise provided in the app?
11. Knowing that balance exercises are recommended for at least 2 hours a week, would you prefer to use the StandingTall app alone, or use it to complement a group exercise program?
12. How many days/minutes a week could you engage with the app?
13. How do you think people of your age in your surroundings would appreciate this option as a way to be more active?
14. What kind of support or resources would you need to use the StandingTall app effectively?
15. Do you have any additional comments regarding the app?
