# Supplementary material for: Understanding the Prospective Acceptability of a Fall Prevention Exercise App Among Canadians Aged 50 Years and Older Living in the Community: Mixed Methods Study
Source: JMIR Form Res. 2026 Aug 13;10:e95812. doi: 10.2196/95812 (PMC13472487; doi:10.2196/95812)
Supplement: Supplementary file 3 [file formative-v10-e95812-s003.docx]

**Appendix 3**

**List of codes**

| **Preliminary list of codes** | **Final list of codes** |
| --- | --- |
| Barriers to app | Barriers to app |
| Barriers to exercise more | Barriers to exercise more |
| Confidence | Confidence |
| Current app use | Current app use |
| Current Exercise tech | Current exercise tech |
| Current Exerciser | Current exerciser |
| Engagement in community exercise | Engagement community exercise |
| Exercise History | Exercise History |
| Exercise History tech | Exercise history tech |
| Feed-back /progress | Fit with lifestyle |
| Fit with lifestyle | Health goals |
| Health goals | Interest |
| Interest | Perceived acceptance by others |
| Perceived acceptance by others | Perceived benefits |
| Perceived benefits | Perceived needs |
| Perceived need | Social Influence |
| Social influence | StandingTall app characteristics |
| Standing Tall app characteristic | Tech Issues |
| Tech issues | Tech use in general |
| Tech use in general | Understanding the app |
